# Affective dynamics following immersive art in a Chinese context: a secondary analysis of pre-post PANAS data

**DOI:** 10.3389/fpsyg.2026.1941264

**Published:** 2026-10-14

**Authors:** Li Gong, Xiaohui Li

**Affiliations:** 1 Academy of Fine Art and Design, Xuchang University, Xuchang, Henan, China; 2 School of Education, Xuchang University, Xuchang, Henan, China

**Keywords:** esthetic experience, Bayesian estimation, dialectical affect, immersive art, PANAS, positive and negative affect, reliable change

## Abstract

**Background:**

Immersive and new-media art is increasingly promoted as a low-cost and scalable route to mood improvement, yet most evidence rests on simple pre–post mean comparisons that ignore measurement error, response shift, and individual variability.

**Methods:**

This study reports a secondary analysis of a within-subjects dataset in which 30 adults in a Chinese setting completed the full 20-item Positive and Negative Affect Schedule (PANAS) immediately before and after an immersive art exposure, with no missing data. We tested parcel-level longitudinal measurement invariance, fitted random-intercept mixed models, classified individual-level reliable change, estimated effects within a Bayesian framework, and compared the positive–negative affect association before versus after the exposure.

**Results:**

Invariance held at the configural and metric levels, ruling out response shift. Negative affect declined by about three points (*b* = −3.13, *p* = 0.013), a small-to-moderate standardized effect (dz = −0.45, 95% CI [−0.96, −0.08]), whereas positive affect did not change reliably (*b* = 0.63, *p* = 0.492). Individual-level classification indicated that 30 percent of respondents showed reliable reductions in negative affect. Bayesian estimation gave weak-to-moderate evidence for a reduction in negative affect (BF10 = 3.19) and moderate evidence against change in positive affect (BF01 = 4.40). The association between positive and negative affect was close to zero before the exposure and did not change afterward (*Z* = −0.27, *p* = 0.785).

**Conclusion:**

The findings suggest that immersive art primarily dampens negative affect while leaving positive affect and the decoupling of the two dimensions intact. This pattern is compatible with, though not confirmatory of, dialectical accounts of affect in East Asian samples, given the inherent orthogonality of the PANAS scales and the absence of a Western comparison group. Implications for the social and individual bases of aesthetic experience are articulated, emphasizing person-contingent responding and culturally valued calm. Tentative implications for arts-in-health practice are discussed, alongside the limitations of a small single-group design without a control condition or demographic covariates.

## 1 Introduction and literature review

Interest in the arts as a resource for mental health has grown quickly over the past decade, and immersive and new-media art now sits at the center of that interest. Unlike a painting on a wall, an immersive installation surrounds the viewer with projected or virtual imagery, sound, and sometimes touch, so that attention is drawn away from ordinary concerns and toward a single enveloping experience. Viewed through the lens of environmental psychology, such a space can be read as a constructed restorative environment. Restorative Environmental Theory and the related Attention Restoration Theory hold that settings which provide a sense of being away, soft fascination, extent, and compatibility allow depleted attentional and affective resources to recover (Kaplan, 1995). An enveloping art installation supplies several of these qualities through its sensory design, which offers a principled reason to expect an effect on affect and situates the present study within the scope of environmental psychology rather than treating the art exposure as a black box. Advocates argue that such experiences can shift mood at low cost and at scale, since a single installation can serve a continuous stream of visitors without a trained therapist present for each one (Godley et al., 2025). Early field studies report encouraging changes in self-reported affect after immersive exposure, including reductions in negative affect and, less consistently, increases in positive affect (Hadavi et al., 2022; Dehove et al., 2025). The promise is real, but the evidence base is still thin and methodologically uneven.

A recurring weakness is the reliance on a single pre-post comparison of group means. This design answers only one question, namely whether the average score moved, and it answers even that question crudely. It says nothing about whether the change exceeds measurement error for any given person, whether the instrument measured the same construct in the same way before and after, or whether the intervention altered the relationship between different components of affect. A systematic review of art viewing and wellbeing found convergent support for reflective and eudaimonic outcomes but a mixed and unsettled picture for hedonic mood (Trupp et al., 2025). Part of that inconsistency likely reflects the coarseness of the analyses rather than genuine instability in the phenomenon. Better questions require better estimation.

Cultural context adds a further layer that the dominant designs miss. In East Asian settings affect is often understood dialectically, so that positive and negative feelings are seen as coexisting rather than as opposite ends of a single dimension (Miyamoto et al., 2010; Spencer-Rodgers et al., 2010). Chinese respondents tend to report more mixed emotional states than North American respondents across many affective contexts, and this tendency has been linked to a broader tolerance of contradiction (Li and Hu, 2022). Whether this looser coupling also holds specifically for affect measured before and after art viewing, rather than for affect in general, has not been established. If positive and negative affect are more loosely coupled in a Chinese sample, then an intervention might move one dimension without moving the other, and it might leave the coupling itself untouched. A study that only reports two mean scores cannot detect any of this. To our knowledge, whether an immersive art exposure changes the coupling between positive and negative affect has not been tested in either a Western or an East Asian sample, so examining it here is both a methodological and a cultural contribution. Any cultural reading, however, remains provisional in the absence of a Western comparison group.

The measurement instrument matters as much as the design. The Positive and Negative Affect Schedule is the most widely used self-report measure of affect, and its two-factor structure has been examined repeatedly, including in Chinese samples where an orthogonal two-factor solution has fit well (Kim and Wang, 2022; Liu et al., 2020). Yet applied wellbeing studies rarely check whether that structure is stable across the two occasions being compared. If the meaning of the items shifts between pre and post, an apparent change in scores may reflect a change in the measurement rather than a change in the person, a problem known as response shift. Ruling out response shift before interpreting mean change is a basic requirement that immersive-art studies almost never meet.

The present study addresses these gaps through a secondary analysis of a within-subjects dataset in which 30 adults in a Chinese setting completed the full 20-item PANAS immediately before and after an immersive art exposure. The contribution is threefold. First, the analysis moves beyond a lone paired test to a triangulated strategy that combines mixed models, individual-level reliable change, latent change estimation, and Bayesian inference, so that a small or absent effect is characterized honestly rather than obscured. Second, it tests longitudinal measurement invariance so that any observed change is interpreted only after response shift has been ruled out. Third, it examines whether the intervention alters the coupling between positive and negative affect, connecting the analysis directly to the dialectical understanding of affect in a Chinese context. The aim is not to inflate a modest dataset into a dramatic finding, but to extract from it every defensible conclusion and to report the uncertainty around each one. To our knowledge this is the first secondary analysis of immersive-art PANAS data in a Chinese context that gates its interpretation of mean change on a formal longitudinal measurement-invariance test, that reports individual-level reliable change alongside the group effect, that estimates the effect in a Bayesian framework with an explicit region of practical equivalence and a Bayes factor for the null, and that directly tests whether the intervention changes the coupling between positive and negative affect.

### 1.1 Immersive and new-media art and wellbeing

The first stream of evidence concerns immersive and new-media art as a wellbeing intervention. A campus study exposed students to a fully immersive projection environment before final examinations and reported reductions across most negative affect items and increases across most positive affect items on the PANAS (Godley et al., 2025). During the pandemic, a randomized comparison of a virtual art exhibit found increases in positive affect and reductions in negative affect after viewing (Hadavi et al., 2022). Repeated visits to a virtual art gallery likewise raised wellbeing, immersion, and esthetic emotion relative to reading about art (Cotter et al., 2024). A laboratory study of urban art found that both art and greenery reduced stress and negative mood, while positive mood remained stable, suggesting that the benefit of art may operate more strongly on the negative side of affect (Dehove et al., 2025). Reviews of virtual-reality art specifically point to promising but still preliminary effects on emotion regulation and restoration (Hadjipanayi et al., 2023), and commentators have highlighted its value for health communication (Shi and Wang, 2024). Across this stream the pattern that recurs is a clearer effect on negative affect than on positive affect, a pattern that the present analysis is well placed to examine.

New-media art in Chinese settings is a small but growing part of this stream. It is important to distinguish two activities that are often grouped together. Art therapy and art making are active processes in which a person creates work and makes material choices, whereas art viewing is a receptive, interpretive process; the two are not equivalent, and evidence from one does not transfer directly to the other. A virtual-reality art therapy system built around classical Chinese poetry produced pre-post gains in self-reported positive emotion and reductions in negative emotion, corroborated by physiological markers (Jin et al., 2025), and qualitative work with Chinese university students describes digital art making as an embodied route to emotional expression and regulation (Xie, 2025). These are art-making studies, and their effects should not be read as direct evidence about art viewing. The form of the art also matters. Classical Chinese poetry is a traditional, contemplative form, whereas immersive new-media art can be playful, political, or deliberately unsettling, so effects observed for one form need not hold for another. Broader reviews of virtual-reality art therapy report promising but preliminary effects on emotion and restoration (Hadjipanayi et al., 2023). Taken together, this work shows that immersive and digital art is being actively deployed for wellbeing in China, which makes rigorous secondary analysis of a Chinese art-viewing dataset timely, while cautioning against treating the different forms and modes of engagement as interchangeable.

### 1.2 Arts in health and museum wellbeing

The second stream places immersive art within the broader arts-in-health evidence base. A meta-analysis of arts-on-prescription programs found small to moderate improvements in wellbeing, with benefits accumulating over repeated engagement (Jensen et al., 2024). An experimental study of physician-prescribed museum visits reported measurable mental-health benefits relative to a comparison condition (Stellar et al., 2026). Research on museum visiting links the experience of awe and esthetic emotion to gains in subjective wellbeing and even to physiological markers of health (Stellar et al., 2026). Curatorial scholarship has begun to treat awe as something that can be deliberately designed into exhibition spaces (Varutti, 2026). Work cataloging the vocabulary of esthetic emotion shows that awe, being moved, and wonder are the terms researchers most consistently treat as esthetic emotions (Schubert, 2024). This stream frames immersive art as one intense instance of a more general mechanism by which cultural engagement supports wellbeing.

### 1.3 Measurement and cross-cultural structure of the PANAS

The third stream concerns the measurement of affect itself. The PANAS was developed to capture two broad and largely independent dimensions of affect, and its construct validity and normative properties have been documented in large non-clinical samples (Watson et al., 1988). In Chinese samples the two-factor structure has been supported, with a Bayesian structural equation analysis of nearly nine hundred students favoring an orthogonal two-factor model (Kim and Wang, 2022). A short-form Chinese version has shown acceptable reliability and measurement invariance across gender and grade (Liu et al., 2020). The dialectical-affect literature adds that positive and negative affect co-occur more readily in East Asian than in North American samples, a difference attributed to dialectical thinking and a greater tolerance of contradiction (Miyamoto et al., 2010; Spencer-Rodgers et al., 2010). Recent work continues to document culturally patterned differences in how positive and negative feelings combine in everyday life (Zhou et al., 2024), and links dialectical thinking to distinct patterns of affective and even neural response (Li and Hu, 2022). Taken together, this stream implies that the coupling between positive and negative affect is itself a meaningful, culturally sensitive quantity worth measuring.

### 1.4 Modern methods for within-person pre-post change

The fourth stream concerns how change should be analyzed. Effect sizes for paired designs, such as the standardized mean difference of the paired scores, should be reported with confidence intervals rather than as bare test statistics (Lakens, 2013; Goulet-Pelletier and Cousineau, 2018). At the individual level, the Reliable Change Index and the Jacobson-Truax framework classify each person as reliably improved, unchanged, or deteriorated relative to measurement error (Jacobson and Truax, 1991), although recent critical appraisals stress that the index is easily misapplied and should be used with care and with explicit reliability assumptions (McAleavey, 2024). Latent change score models recover true change at the construct level while accounting for measurement error, and they can be estimated with as few as two occasions (Cáncer and Estrada, 2023; Wiedemann et al., 2022; Cáncer et al., 2021). Bayesian estimation complements these tools by expressing conclusions as posterior distributions, by using a region of practical equivalence to decide whether an effect is negligible, and by quantifying evidence for the null through a Bayes factor (Makowski et al., 2019). The present analysis draws on all four tools so that its conclusions do not depend on any single method. Table 1 sets these strands side by side, summarizing the setting, sample, design, affect measure, analytic method, and principal finding of representative studies from all four streams and locating the present study within them.

**Table 1:** Summary of representative studies on art exposure and affective wellbeing.

Study	Setting / Sample	Design	Affect measure	Analysis	Principal finding
Godley et al. (2025)	US campus immersive projection; students	Single-group pre-post	PANAS-SF	Wilcoxon signed-rank	Negative affect fell, positive affect rose after exposure
Hadavi et al. (2022)	Virtual art exhibit during COVID-19	Randomized pre-post	PANAS	Group mean comparison	Positive affect increased, negative affect decreased
Dehove et al. (2025)	Urban art laboratory; adults	Controlled pre-post	Mood and stress scales	Mixed ANOVA	Stress and negative mood fell; positive mood unchanged
Trupp et al. (2025)	Museum and gallery art viewing	Systematic review	Various wellbeing scales	Narrative synthesis	Eudaimonic gains consistent; hedonic mood gains mixed
Jensen et al. (2024)	Community arts on prescription	Meta-analysis	WHO-5 and related	Random-effects meta-analysis	Small to moderate wellbeing improvement
Kim and Wang (2022)	Chinese students; *N* = 893	Cross-sectional	PANAS	Bayesian SEM	Orthogonal two-factor structure fit best
Present study	Chinese immersive art; *N* = 30	Single-group pre-post	Full 20-item PANAS	Mixed models, RCI, LCS, Bayesian, coupling test	Negative affect fell; positive affect and affect coupling stable

PANAS, Positive and Negative Affect Schedule; SF, short form; RCI, Reliable Change Index; LCS, latent change score; SEM, structural equation modeling.

### 1.5 Research gap

Three gaps emerge from these streams. Immersive-art wellbeing studies are dominated by simple pre-post mean comparisons that ignore measurement error, response shift, and individual-level meaningful change. Almost none test whether the measurement structure of the affect instrument is stable across the two occasions being compared, so apparent change may be confounded with response shift. And almost none ask whether the intervention alters the coupling between positive and negative affect, even though that coupling is theoretically central in a Chinese sample. The present study targets all three gaps directly.

## 2 Hypotheses

Four expectations follow from the literature. The first concerns positive affect. Because prior work finds inconsistent and often null effects of art exposure on positive mood, the expectation is deliberately weak: positive affect may increase modestly or may not change (H1). The second concerns negative affect. Because the clearest and most replicated effect of art exposure is on the negative side, negative affect is expected to decrease from pre to post (H2). The third concerns individual change. A nontrivial proportion of respondents is expected to show reliable improvement in negative affect that exceeds measurement error, rather than a uniform shift across everyone (H3). The fourth concerns the affect coupling. Given the dialectical understanding of affect in a Chinese sample, positive and negative affect are expected to be weakly associated at baseline, and the intervention is not expected to strengthen that coupling; whether it weakens it further is treated as an open question (H4).

## 3 Dataset

The data are a within-subjects pre-post dataset drawn from the Science Data Bank (SciDB) repository (Cen et al., 2024). Thirty adults in a Chinese setting completed the full 20-item Positive and Negative Affect Schedule immediately before and immediately after an immersive art exposure. According to the repository record, the exposure was an interactive art installation that integrated virtual reality (VR), electroencephalography (EEG), and artificial-intelligence-generated content (AIGC), designed by the original investigators to alleviate negative emotions. The available data document the affective measurements rather than the fine sensory and technical parameters of the installation, so variables such as illuminance, sound level, color temperature, spatial scale, and exposure duration were not recorded and cannot be analyzed here; this absence is noted as a constraint of the secondary data. Each respondent is identified by a stable label that appears at both occasions, which confirms that the design is genuinely within-subjects rather than two independent samples. The instrument comprises 10 positive affect items (interested, excited, strong, enthusiastic, proud, inspired, determined, attentive, active, alert) and 10 negative affect items (irritable, ashamed, nervous, jittery, afraid, guilty, scared, hostile, upset, distressed), each rated on a five-point scale from 1 (very slightly or not at all) to 5 (extremely). The achieved sample size is 30, both occasions are complete, and there are no missing responses in any of the 40 item columns. An earlier study that combined the EEG recordings in the same repository with PANAS change scores described the participant pool as 50 university students and likewise reported a reduction in negative affect without a reliable change in positive affect (Zeng and Chen, 2025); the results reported here refer to the 30 respondents in the item-level PANAS file analyzed in the present study. The PANAS data file contains item responses and identifiers only; no demographic covariates such as age, sex, or exposure dose are recorded in it, so covariate-adjusted and subgroup analyses are not possible and are not attempted here. The repository also holds EEG recordings taken before and after the exposure; these lie outside the scope of the present analysis, which uses the PANAS responses only.

## 4 Data preparation

Preparation followed a fixed sequence appropriate to survey data. The item responses were loaded and the respondent identifiers were matched across the two occasions to confirm that every person present at pre was also present at post. The response values were screened against the permitted range of one to five, and none fell outside it. Careless and straight-line responding was checked by computing, for each respondent at each occasion, the standard deviation of their 20 item responses; the smallest within-person standard deviation was 0.44 at pre and 0.57 at post, so no respondent answered with a single repeated value and none was excluded on quality grounds. Because the dataset contained no missing values, no missing-data mechanism had to be assumed and no imputation was performed; had missingness been present, a missing-at-random assumption with full-information maximum likelihood would have been the default. None of the 20 PANAS items is reverse keyed, so no reverse scoring was required. Positive and negative affect composites were then formed as the sum of their 10 constituent items, giving four composite scores per respondent that range from 10 to 50. Item-level means and standard deviations for both occasions are reported in Table 2.

**Table 2:** Item-level means and standard deviations at pre and post occasions (*N* = 30).

Item	Pre M	Pre SD	Post M	Post SD
Positive affect items				
Interested	3.17	0.75	3.53	0.82
Excited	2.83	0.91	3.23	1.01
Strong	3.00	1.02	2.53	1.04
Enthusiastic	2.70	0.79	3.03	0.89
Proud	2.37	0.81	2.33	0.88
Inspired	2.60	1.00	2.80	1.06
Determined	2.57	0.94	2.33	1.03
Attentive	3.33	0.76	3.57	0.97
Active	3.00	0.95	3.10	0.88
Alert	2.03	0.93	1.77	0.94
Negative affect items				
Irritable	1.93	1.14	1.57	0.86
Ashamed	1.43	0.77	1.17	0.38
Nervous	2.37	0.96	1.90	0.99
Jittery	2.03	1.13	1.70	0.95
Afraid	1.53	0.78	1.53	0.78
Guilty	1.53	0.78	1.33	0.61
Scared	1.60	0.93	1.53	0.82
Hostile	1.50	0.90	1.23	0.50
Upset	2.07	1.05	1.47	0.78
Distressed	2.00	1.08	1.43	0.77

Responses on a 1 (very slightly or not at all) to 5 (extremely) scale.

For the structural equation models, three balanced parcels were constructed within each 10-item subscale by assigning items in rotation, which yields parcels of comparable content and improves the ratio of cases to estimated parameters. Reliability was computed for each subscale at each occasion using both Cronbach alpha and McDonald omega. Internal consistency was high throughout, with alpha ranging from 0.875 to 0.931 and omega ranging from 0.902 to 0.947, as reported in Table 3. These values indicate that the composites are measured reliably enough to support the change analyses that follow. The item-level statistics in Table 2 show that the negative affect items sat near the low end of the scale and drifted lower after the exposure while the positive affect items barely moved.

**Table 3:** Descriptive statistics and internal-consistency reliability for the affect composites.

Scale / Occasion	M	SD	α	ω	Skew
Positive affect, pre	27.60	6.11	0.875	0.902	0.04
Positive affect, post	28.23	6.71	0.886	0.910	−0.01
Negative affect, pre	18.00	7.55	0.931	0.944	1.19
Negative affect, post	14.87	6.00	0.930	0.947	1.40

α = Cronbach alpha; ω = McDonald omega. Composites are the sum of 10 items and range from 10 to 50.

## 5 Exploratory analysis

Exploratory analysis characterized the distributions before any model was fitted. Item-level and composite distributions were inspected for ceiling and floor effects. Negative affect items clustered near the low end of the scale, as is typical for a non-clinical sample, but none was so compressed that variance vanished. The missing-data map in Figure 1 confirms visually what the numeric check established, namely that every cell is populated. Figure 2 shows the paired distributions of the two composites as raincloud plots, with each respondent connected across occasions; the negative affect cloud shifts downward from pre to post while the positive affect cloud barely moves. Figure 3 plots positive against negative affect at each occasion and shows that the two dimensions are almost unrelated at both times. Figure 4 presents the correlation matrix of the four composites, which shows pre-post stability within each dimension (*r* = 0.69 for positive affect and *r* = 0.50 for negative affect) and near-zero concurrent associations between the two dimensions at each occasion (*r* = 0.05 before and *r* = 0.11 after); the one sizeable cross-occasion value, between positive affect before and negative affect after the exposure (*r* = 0.50), is reported descriptively and was not tested. Together the exploratory displays anticipate the modeling results: a downward shift in negative affect, a stable positive affect, and a persistent decoupling of the two dimensions.

**Figure 1:**
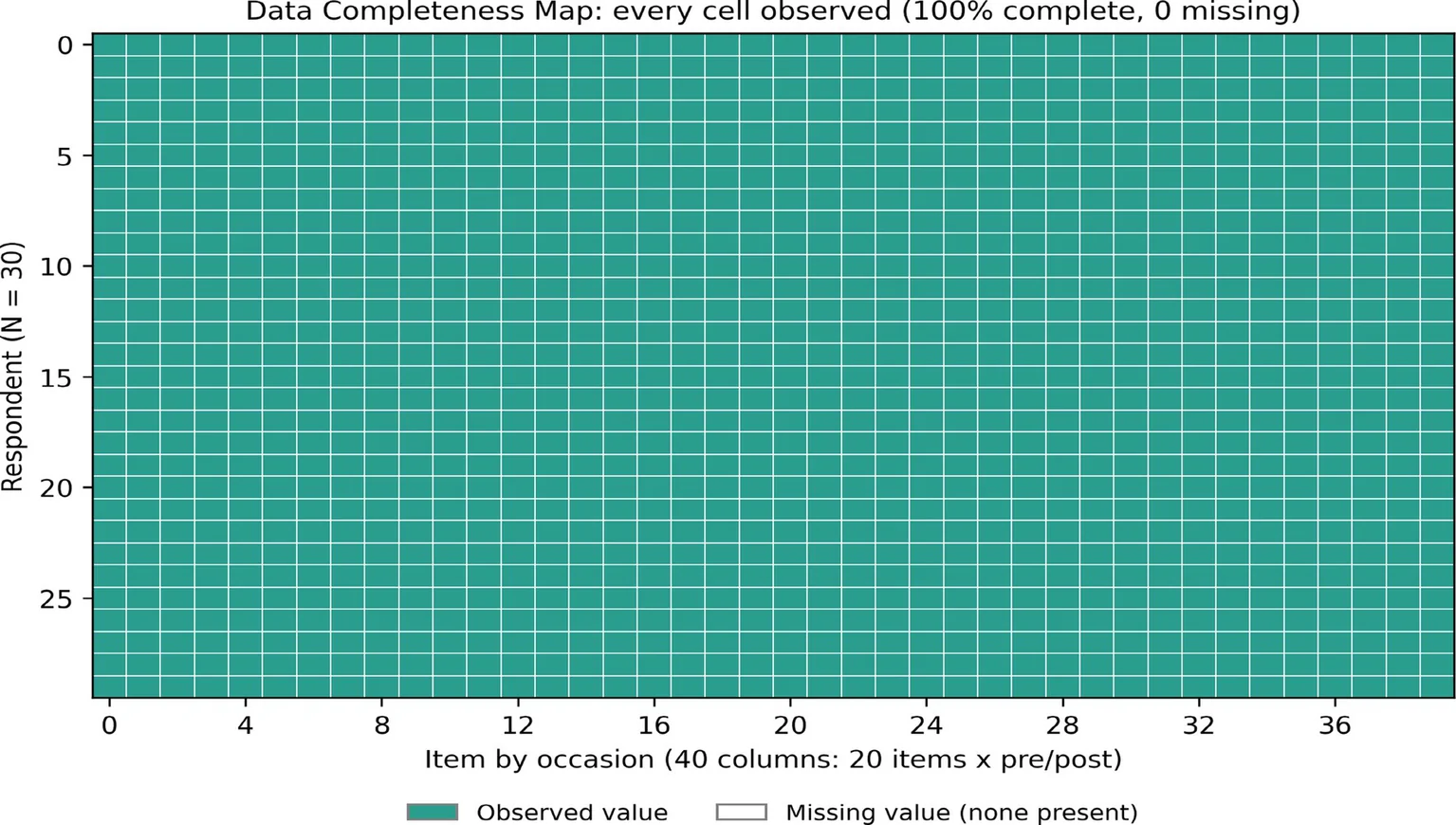
Missing-data map across respondents and item-by-occasion columns. No missing values were observed.

**Figure 2:**
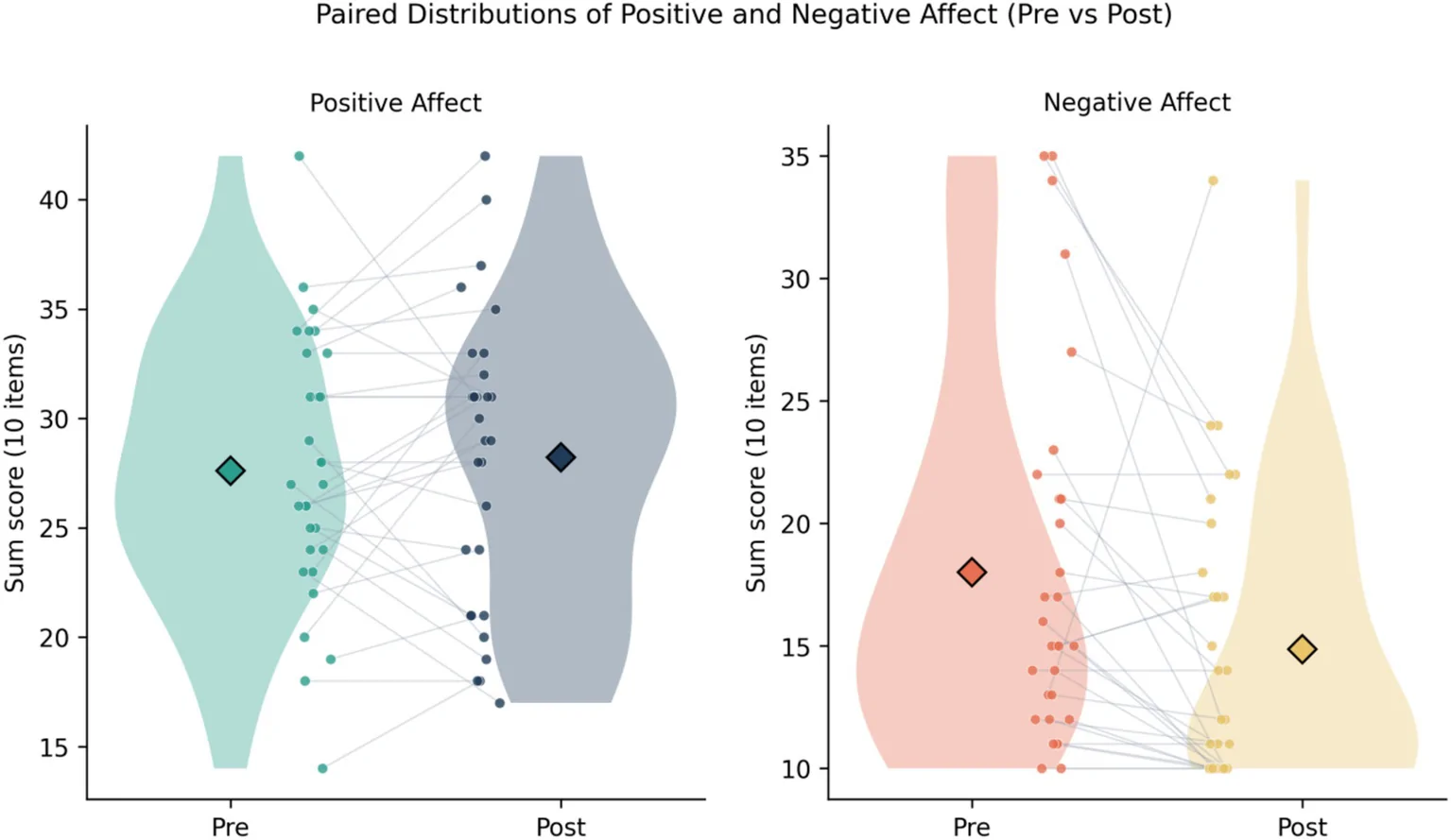
Paired raincloud distributions of positive and negative affect composites at pre and post. Diamonds mark occasion means; thin lines connect the same respondent.

**Figure 3:**
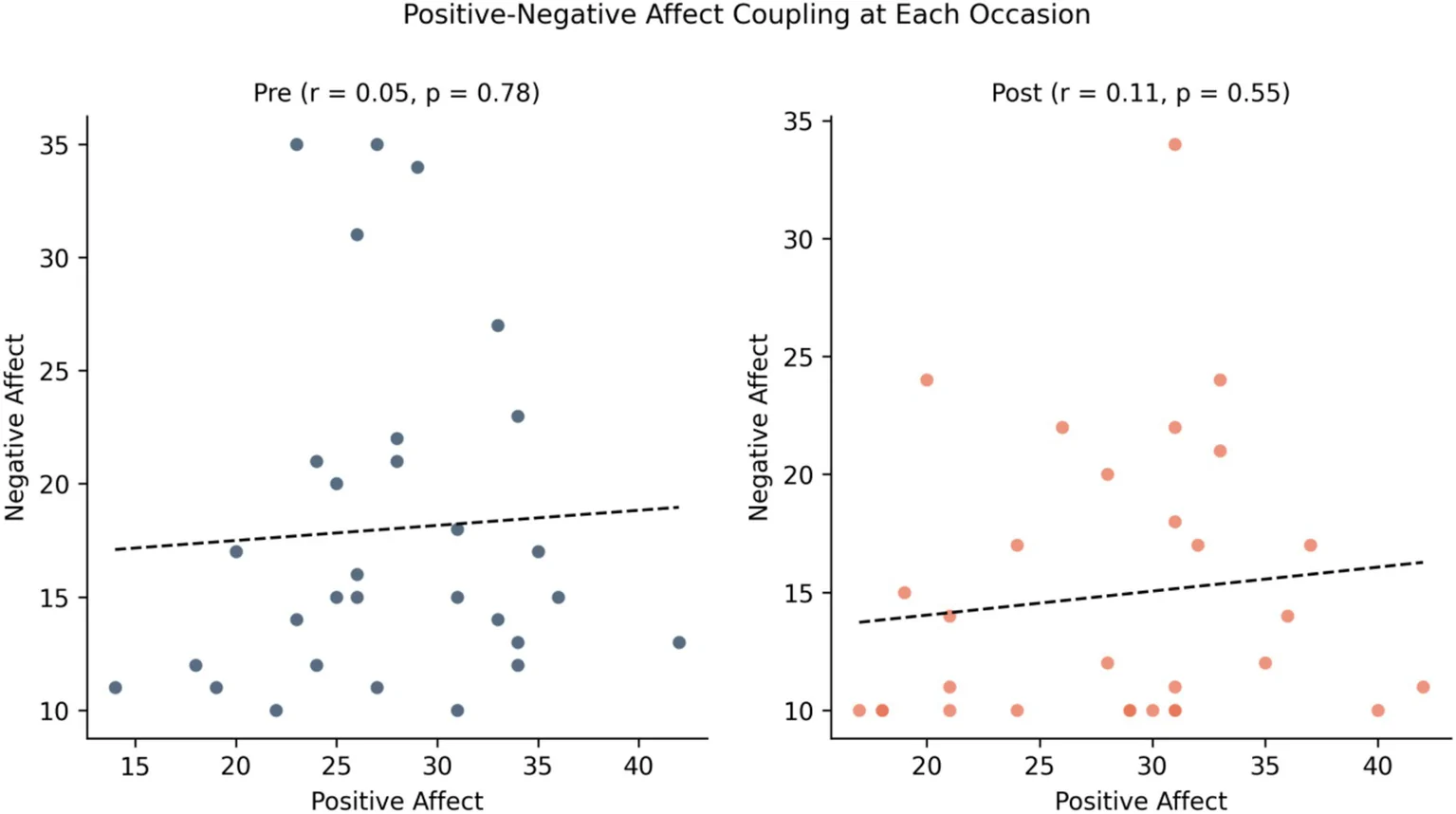
Positive against negative affect at each occasion, with the fitted regression line and the correlation. The two dimensions are nearly independent at both times.

**Figure 4:**
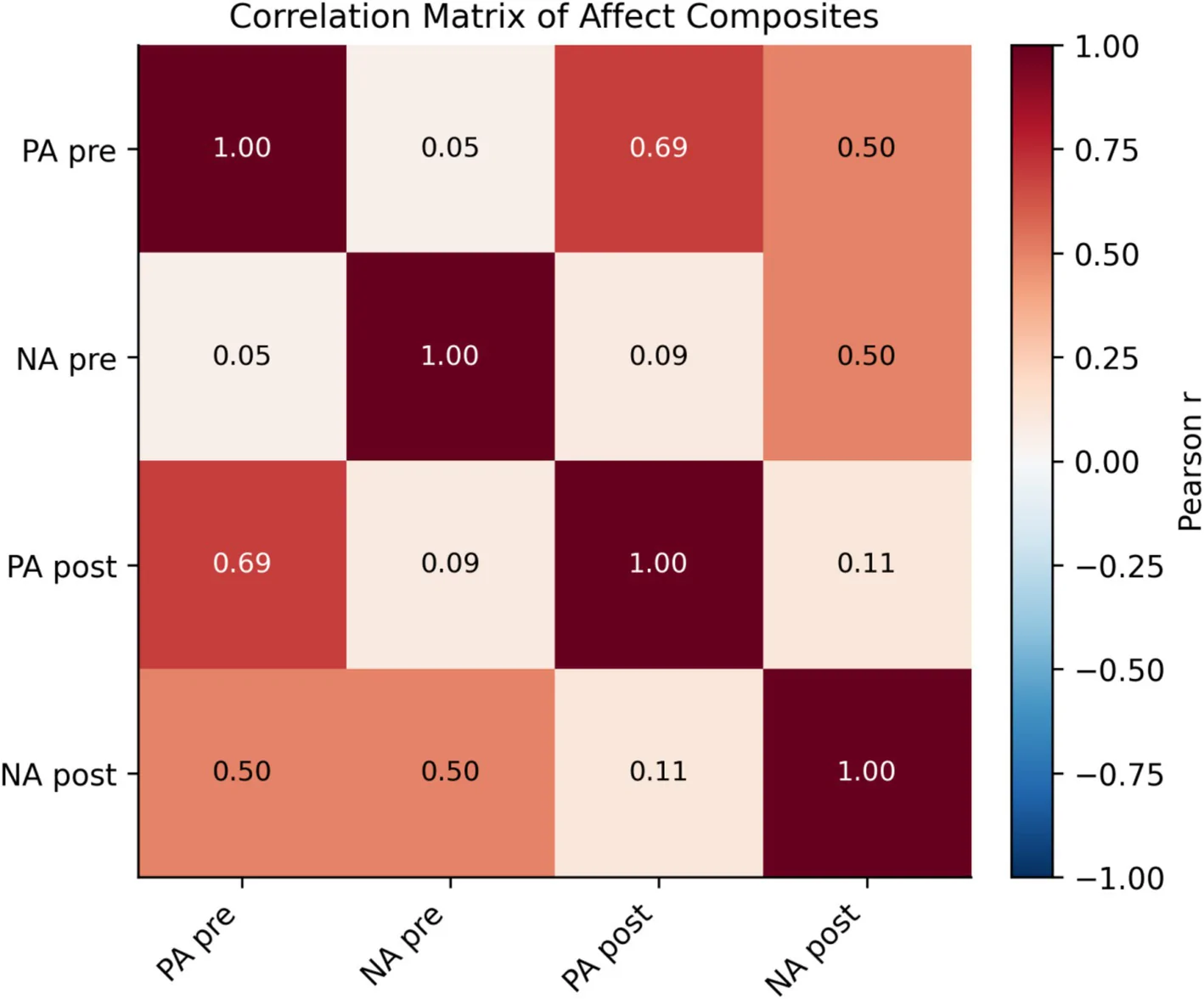
Correlation matrix of the four affect composites, showing within-dimension stability across occasions and near-zero concurrent coupling between the two dimensions at each occasion.

## 6 Psychometric analysis

The psychometric analysis served a gatekeeping function: mean change should be interpreted only after the measurement structure is shown to be stable. Internal consistency, reported above, was high for both scales at both occasions. Confirmatory models were then estimated at the parcel level using maximum-likelihood estimation, with three parcels loading on each latent factor and correlated residuals linking the same parcel across occasions. A longitudinal measurement-invariance sequence was fitted for each scale. The configural model allowed all loadings to differ across occasions, and the metric model constrained the loadings to be equal across occasions. Invariance was judged by the change in the comparative fit index, with a change no larger than 0.01 in magnitude taken as evidence that the constraint held.

The results appear in Table 4. For positive affect the configural model fit well (CFI = 0.977), and imposing equal loadings did not worsen fit (ΔCFI = 0.010), so metric invariance held. For negative affect the pattern was the same, with a negligible change in fit when loadings were constrained (ΔCFI = −0.001). The root mean square error of approximation was elevated for some models, which is expected when the sample is small and the degrees of freedom are few, and it is reported transparently rather than suppressed. Because the loadings were invariant across occasions, the observed change in scores can be attributed to change in the underlying affect rather than to a shift in how the items relate to the construct. This licenses the interpretation of the change models that follow. Full scalar invariance was not separately imposed given the sample size, and this limitation is acknowledged.

**Table 4:** Longitudinal measurement-invariance tests at the parcel level.

Model	χ^2^	df	CFI	RMSEA	ΔCFI
Positive affect, configural	8.25	5	0.977	0.150	n/a
Positive affect, metric	8.89	7	0.987	0.096	0.010
Negative affect, configural	16.41	5	0.941	0.281	n/a
Negative affect, metric	18.55	7	0.941	0.239	−0.001

Configural and metric models estimated on three balanced parcels per factor per occasion with correlated residuals across occasions. A ΔCFI no larger than 0.01 in magnitude indicates invariance holds. Scalar models are not reported because parcel means were not separately constrained given the sample size.

## 7 Analytic pipeline

The analytic core combined four complementary approaches so that no conclusion depends on a single method. These approaches are not four independent findings but convergent checks on the same small dataset; given the sample size, they are reported to gauge the robustness of one modest effect rather than to multiply claims, and the more data-hungry models are read as indicative only. All analyses were conducted in Python. Mixed models were estimated with statsmodels, effect sizes and correlations with pingouin, structural equation models with semopy, and Bayesian models with PyMC. A fixed random seed of 20,260,716 was used throughout for reproducibility. The pipeline is summarized in Figure 5 and specified step by step in Algorithm S1.

**Figure 5:**
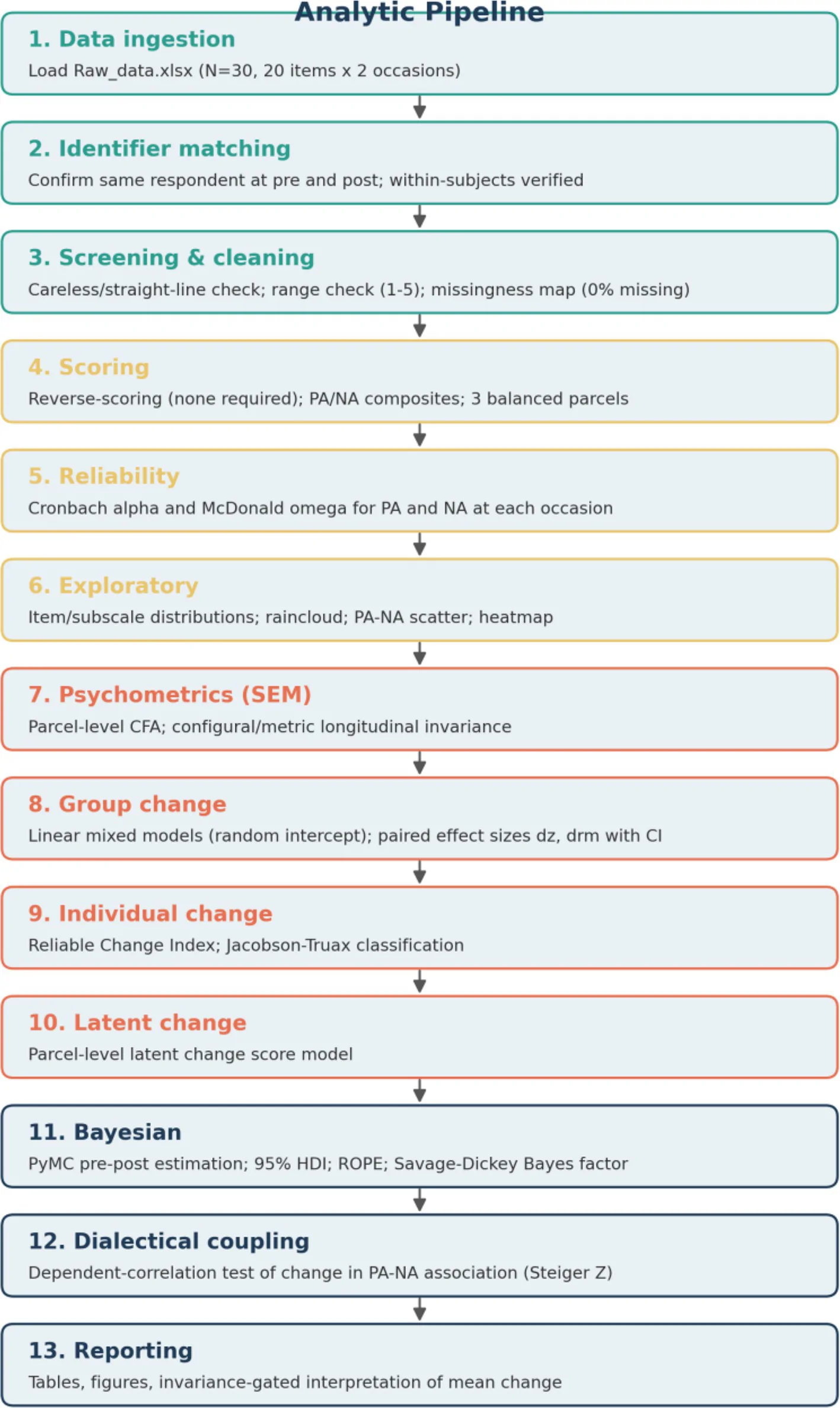
Analytic pipeline from data ingestion through invariance-gated interpretation of change.

The full pseudocode for this pipeline is provided as Algorithm S1 in the Supplementary Materials. It reads from top to bottom. The first block prepares the data by loading the responses, matching identifiers across the two occasions, screening the scale range, flagging straight-line responding, forming the two composites, and building the parcels. The second block establishes measurement quality by computing reliability and by fitting the configural and metric invariance models for each scale, gating everything that follows on a change in the comparative fit index no larger than 0.01 in magnitude. The third block estimates change four ways for each affect dimension: a mixed model for the group effect, standardized effect sizes with bootstrap intervals, the Reliable Change Index with Jacobson-Truax classification for individual change, a parcel-level latent change score model for true change, and a Bayesian posterior with a region of practical equivalence and a Bayes factor. The final step tests whether the exposure changed the coupling between positive and negative affect through a dependent-correlation statistic. Each numbered line in the algorithm corresponds directly to a quantity reported in the results.

### 7.1 Equations

The random-intercept mixed model for an outcome is given in Equation 1, where *Y*_ij_ is the composite score for respondent *i* at occasion *j*, *β*_0_ is the intercept, *β*_1_ is the fixed effect of occasion coded zero for pre and one for post, *u*_0i_ is the respondent random intercept, and *ε*_ij_ is the residual.

$$Y_{\mathrm{ij}}=\beta_{0}+\beta_{1}\;\text{Occasio}n_{\mathrm{ij}}+u_{0i}+\varepsilon_{\mathrm{ij}}$$ (1)

The standardized paired effect size in Equation 2 divides the mean of the paired differences *M*_D_ by their standard deviation *SD*_D_, where *D*_i_ is the post-minus-pre difference for respondent *i* and *n* is the number of respondents.

$$d_{z}=\frac{M_{D}}{SD_{D}}=\frac{\frac{1}{n}\Sigma \;D_{i}}{\sqrt{\frac{\Sigma {\left(D_{i}-M_{D}\right)}^{2}}{n-1}}}$$ (2)

The Reliable Change Index in Equation 3 divides the observed change by the standard error of the difference *S*_diff_, which is defined as the square root of twice the squared standard error of measurement; the standard error of measurement is the pre-occasion standard deviation multiplied by the square root of one minus the reliability. A respondent is classified as reliably changed when the index exceeds 1.96 in the improving or deteriorating direction.

$$\mathrm{RCI}=\frac{X_{\text{post}}-X_{\mathrm{pre}}}{S_{\text{diff}}},\;\;S_{\text{diff}}=\sqrt{2\;S{E^{2}}_{\text{meas}}}$$ (3)

The latent change score specification in Equation 4 defines the post latent factor *η*_post_ as the pre factor *η*_pre_ plus a latent change factor Δη, whose mean *α*_Δ_ captures average true change, whose regression *β*_Δ_ on the baseline captures change-from-baseline coupling, and whose disturbance *ζ*_Δ_ captures individual variation in change.

$$\eta_{\text{post}}=1\cdot \eta_{\mathrm{pre}}+\Delta \eta ,\;\;\Delta \eta =\alpha_{\Delta }+\beta_{\Delta }\;\eta_{\mathrm{pre}}+\zeta_{\Delta }$$ (4)

The Bayesian model in Equation 5 expresses the posterior of the standardized change μ as proportional to the likelihood times weakly informative priors, with a standard normal prior on μ and a half-normal prior on the standard deviation σ. A region of practical equivalence of plus or minus one tenth of a standard deviation defines the band of effects treated as negligible, and a Savage-Dickey Bayes factor quantifies evidence for or against the null.

p(μ∣D)∝p(D∣,μ∣,σ)p(μ)p(σ),ROPE=[−0.1,0.1] (5)

### 7.2 Estimation settings

The mixed models used restricted maximum likelihood with a respondent random intercept and occasion as the sole fixed effect; when the default optimizer failed to converge, a Powell or Nelder–Mead optimizer was substituted. Effect-size confidence intervals came from ten thousand bootstrap resamples of the paired differences. The structural equation models used maximum-likelihood estimation on the parcel covariance structure, and invariance was judged by a change in the comparative fit index no larger than 0.01. The Bayesian models were sampled with four chains of four thousand draws each after two thousand tuning iterations, with a target acceptance probability of 0.95; convergence was assessed by the potential scale reduction factor and the effective sample size. The dependent-correlation test used the Steiger formulation for two correlations with no variable in common. All random seeds were fixed at 20260716.

## 8 Results

### 8.1 Group-level change

The mixed models gave a clear and asymmetric result. Negative affect declined from a mean of 18.00 to 14.87, a fixed effect of occasion of −3.13 points with a 95% confidence interval of [−5.61, −0.66] and *p* = 0.013. Positive affect moved only from 27.60 to 28.23, a fixed effect of 0.63 with a confidence interval of [−1.17, 2.44] and *p* = 0.492, which is indistinguishable from no change. The standardized effect sizes tell the same story. For negative affect dz. was −0.45 with a bootstrap interval of [−0.96, −0.08], a small to moderate effect whose interval excludes zero. For positive affect dz. was 0.13 with an interval of [−0.22, 0.53] that comfortably includes zero. These results support H2 and are consistent with the deliberately weak H1, under which positive affect was allowed to remain unchanged. Table 5 collects the group-level estimates.

**Table 5:** Group-level pre-post change from mixed models, standardized effect sizes, and Bayesian estimation.

Outcome	b (occasion) [95% CI]	dz [95% CI]	Bayesian mean [95% HDI]	BF10
Positive affect	0.63 [−1.17, 2.44]	0.13 [−0.22, 0.53]	0.12 [−0.24, 0.48]	0.23
Negative affect	−3.13 [−5.61, −0.66]	−0.45 [−0.96, −0.08]	−0.44 [−0.79, −0.08]	3.19

b is the fixed effect of occasion (post minus pre) from a random-intercept mixed model. dz is the standardized paired effect size with a bootstrap 95% confidence interval (10,000 resamples). Bayesian estimates are the posterior mean and 95% highest-density interval of the standardized change; BF10 is the Savage-Dickey Bayes factor favoring a nonzero effect. Positive affect mixed-model *p* = 0.492; negative affect *p* = 0.013.

The Bayesian analysis reinforced these conclusions and expressed them as evidence rather than as a binary verdict. For negative affect the posterior mean of the standardized change was −0.44 with a 95% highest-density interval of [−0.79, −0.08], only 3.0 percent of the posterior fell within the region of practical equivalence, and the Bayes factor of 3.19 indicated only weak-to-moderate evidence for an effect, a value that sits near the boundary between anecdotal and moderate support and should not be read as decisive. For positive affect the posterior mean was 0.12 with a wide interval spanning zero, roughly 35 percent of the posterior lay inside the region of practical equivalence, and the Bayes factor favored the null by a factor of 4.40. All chains converged cleanly, with a potential scale reduction factor of 1.000 and effective sample sizes above nine thousand. Figure 6 displays the two posteriors against the region of practical equivalence. The frequentist and Bayesian analyses converge on the same asymmetric pattern, which lends the qualitative conclusion some stability, although the strength of the evidence for the negative-affect reduction remains modest given the small sample.

**Figure 6:**
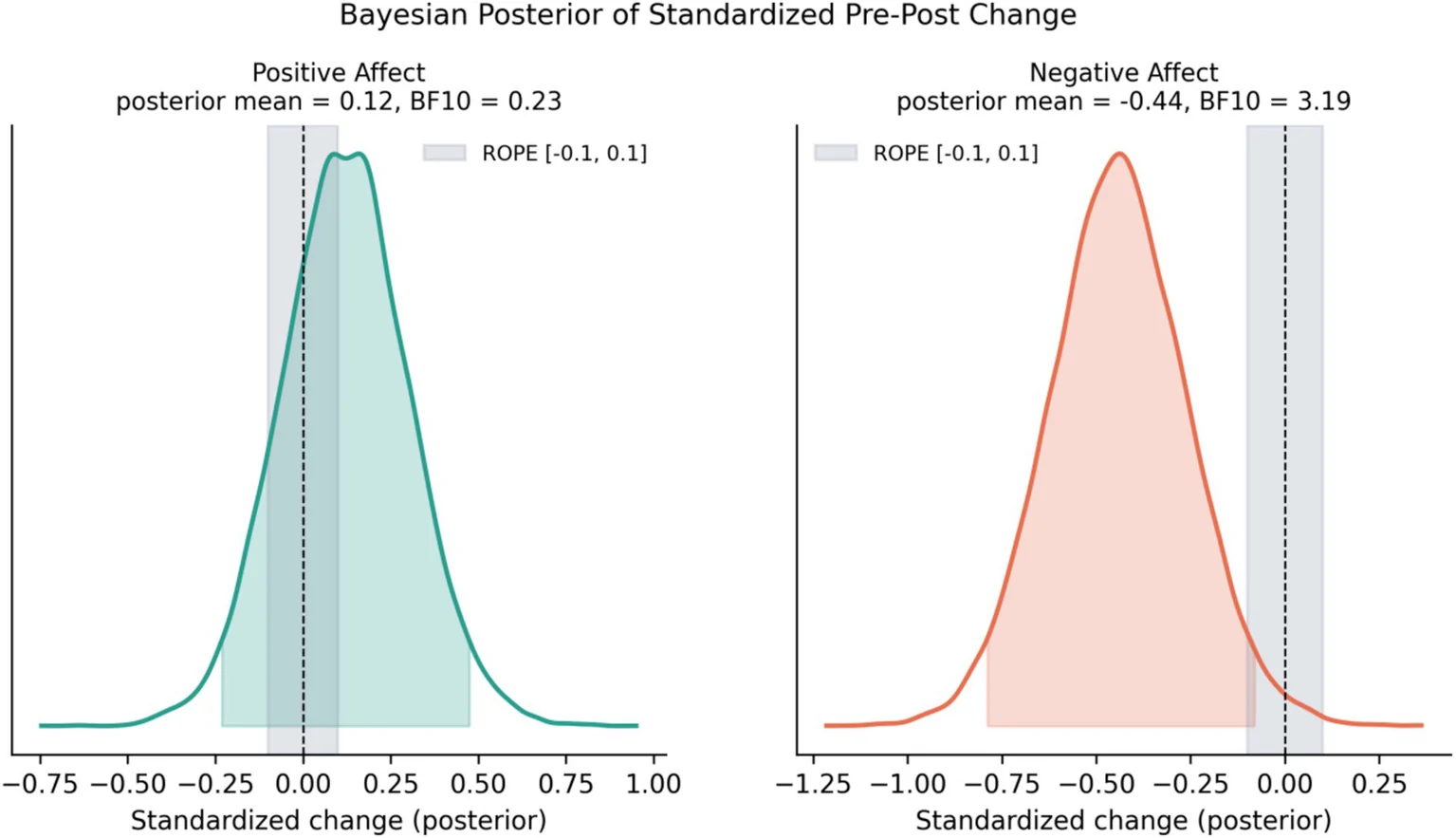
Bayesian posterior distributions of the standardized pre-post change for each affect dimension, shaded within the 95% highest-density interval, with the region of practical equivalence marked.

### 8.2 Individual-level change

Group means can hide the fact that people move in different directions, so individual change was classified with the Reliable Change Index. For negative affect, 9 of the 30 respondents (30 percent) showed a reliable reduction that exceeded measurement error, 20 (67 percent) showed no reliable change, and only 1 (3 percent) showed a reliable increase. For positive affect the movement was more symmetric and smaller, with 4 reliably improved and 4 reliably deteriorated, and the large majority unchanged. This supports H3 and shows that the group-level reduction in negative affect is driven by a substantial minority of clear responders rather than by a uniform nudge across everyone. Figure 7 displays every respondent’s reliable-change score, and Table 6 summarizes the classification.

**Figure 7:**
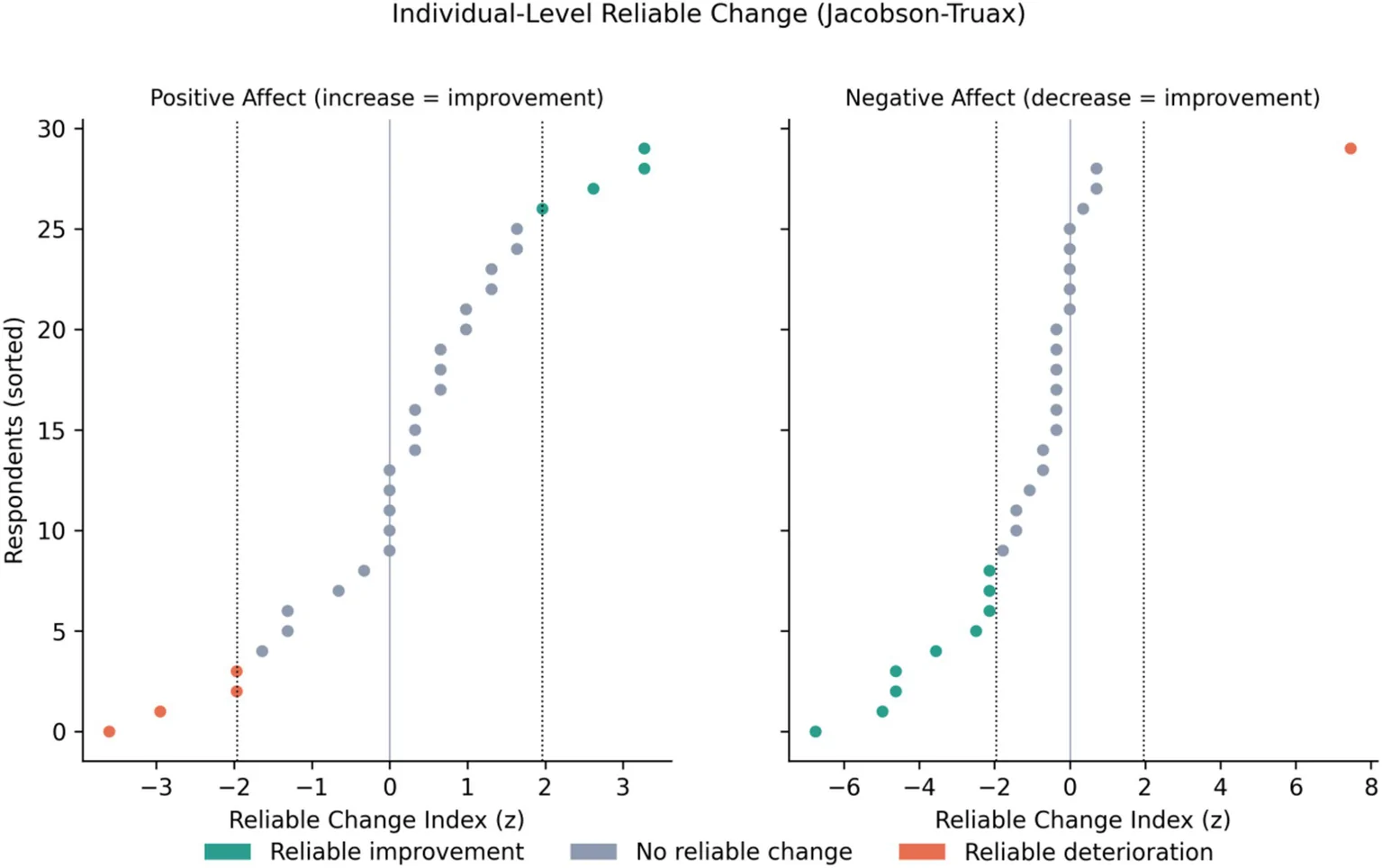
Individual reliable-change scores for each affect dimension. Points beyond the dotted lines at plus or minus 1.96 indicate reliable change.

**Table 6:** Individual-level reliable change classification (Jacobson-Truax).

Outcome	Reliable improvement	No reliable change	Reliable deterioration
Positive affect	4 (13%)	22 (73%)	4 (13%)
Negative affect	9 (30%)	20 (67%)	1 (3%)

Classification uses a Reliable Change Index threshold of ±1.96 with the standard error of measurement derived from the pre-occasion reliability. For positive affect an increase counts as improvement; for negative affect a decrease counts as improvement. S_diff = 3.05 (positive) and 2.81 (negative).

### 8.3 Latent change and affect coupling

The parcel-level latent change score models, which recover true change while accounting for measurement error, were consistent with the composite-level results in direction, showing a downward latent change for negative affect and a near-zero latent change for positive affect. These models are reported with caution because a sample of 30 supports only a simplified parcel-level specification, and their fit indices should be read as indicative rather than definitive. The central cultural question concerned the coupling between positive and negative affect. The two dimensions were essentially uncorrelated before the exposure (*r* = 0.05) and remained so afterward (*r* = 0.11). A dependent-correlation test found no reliable change in this coupling (*Z* = −0.27, *p* = 0.785). This supports H4. The near-zero and stable coupling is a noteworthy descriptive finding. It is consistent with a dialectical organization of affect in which positive and negative feelings operate independently rather than as opposites, but it must be interpreted cautiously. Near-orthogonality of the positive and negative scales is a well-documented property of the PANAS itself, so the present pattern cannot be attributed to culture without a Western comparison group measured under the same conditions. What can be said is that the intervention lowered negative affect without pulling the two dimensions into alignment.

## 9 Discussion

### 9.1 Theoretical implications

Theoretically, the pattern speaks to how affect is regulated in a dialectical cultural frame. If positive and negative affect were strongly opposed, a reduction in negative affect would tend to drag positive affect upward, yet that did not happen here. The independence of the two dimensions, both before and after the exposure, is compatible with accounts in which East Asian affect tolerates the coexistence of opposing feelings (Miyamoto et al., 2010; Spencer-Rodgers et al., 2010), although it is equally compatible with the general psychometric independence of the PANAS scales. Distinguishing these two explanations would require a Western comparison group, which this dataset does not contain. Immersive art in this sample appears to work by soothing the negative dimension rather than by amplifying the positive one, and it does so without collapsing the dialectical structure of affect. This asymmetry fits a restorative-environment reading: an enveloping, softly fascinating space is well suited to lowering tension and negative arousal, which is closer to stress reduction than to the active generation of positive affect (Kaplan, 1995). Because the dataset does not record the physical and sensory parameters of the installation, the specific environmental features responsible cannot be isolated, and identifying them is left to controlled work that manipulates those variables directly. This is a more specific claim than the usual statement that art improves mood, and it is only visible because the analysis modeled the coupling directly.

### 9.2 Implications for the social and individual bases of esthetic experience

Current models in empirical esthetics treat esthetic experience as the joint product of an artwork, a perceiver, and the setting in which they meet. The model of esthetic appreciation and esthetic judgment specifies that an encounter begins with the classification of an object as art in a given context, that the perceiver’s knowledge, personal taste, and affective state shape how the work is processed, and that each encounter yields both an esthetic emotion and an esthetic judgment (Leder et al., 2004). Its later review places this process within an esthetic episode that unfolds over time and gives a central role to context (Leder and Nadal, 2014), and the Vienna Integrated Model of Art Perception adds that the same work can lead different viewers to qualitatively different outcomes, from facile recognition or harmonious pleasure to negative or transformative experiences, depending on how self-relevant the work is and how the viewer resolves discrepancies (Pelowski et al., 2017). Read against these frameworks, the present results speak less to whether immersive art improves mood in general than to three narrower questions: for whom, in what affective form, and under which cultural and social conditions it does so. Because the dataset contains no individual-difference measures, no record of the viewing situation, and no comparison culture, the implications below are stated as grounded propositions for future testing rather than as conclusions.

The first implication concerns the individual basis of the esthetic response. A defining question for the field is how far esthetic experience is shared and how far it is personal, and agreement between observers has been shown to be markedly lower for artworks and architecture than for faces and natural landscapes (Vessel et al., 2018). The present data provide an affective counterpart to this distinction. The direction of change in negative affect was largely shared, since reliable reductions outnumbered reliable increases by nine to one, but its occurrence was not, since two thirds of respondents showed no reliable change (Table 6). Change in positive affect was personal in both occurrence and direction, with four reliable gains matched by four reliable losses. The group-level reduction in negative affect is therefore better described as the aggregate of a responsive minority than as a uniform property of the installation. Individual-difference research identifies plausible sources of this heterogeneity. Openness to experience predicts the tendency to feel awe in response to nature and music (Silvia et al., 2015) and sensitivity to novelty in the esthetic emotions of interest and pleasure (Fayn et al., 2015). In virtual galleries, openness is linked to how much emotion and immersion visitors experience, while immersion, esthetic emotion, extraversion, and neuroticism are associated with the well-being benefits of viewing (Cotter et al., 2024). *In situ* studies of installation art likewise show that viewers’ appraisal strategies and meaning making shape how deeply they engage with the same work (Pelowski et al., 2018). The broader implication for the esthetic mind is that an immersive work does not deliver a fixed dose of affect; it offers an occasion whose affective value depends on what each viewer brings to it.

The second implication concerns what the esthetic response consists of. Esthetic emotions are defined by their direct contribution to esthetic evaluation, with each tuned to a particular type of perceived esthetic appeal (Menninghaus et al., 2019). The terms researchers most consistently use for them include awe, being moved, and wonder (Schubert, 2024), and a dedicated instrument measures prototypical esthetic emotions such as the feeling of beauty, being moved, fascination, and awe alongside epistemic emotions such as interest (Schindler et al., 2017). The null result for the positive affect composite should therefore not be taken as evidence that no positive esthetic response occurred. The item-level means in Table 2, which were not tested inferentially and are described here only to generate hypotheses, show that items describing engaged interest moved upward (interested from 3.17 to 3.53, excited from 2.83 to 3.23, enthusiastic from 2.70 to 3.03, attentive from 3.33 to 3.57, and inspired from 2.60 to 2.80), whereas items describing agency and vigilance moved downward (strong from 3.00 to 2.53, determined from 2.57 to 2.33, and alert from 2.03 to 1.77). Summed into one composite, these opposing movements largely cancel. The pattern is compatible with appraisal accounts in which interest arises when an object is appraised as novel and complex yet comprehensible (Silvia, 2008), and with a receptive mode of engagement in which effortful, goal-directed activation recedes, as the soft fascination of restorative settings would predict (Kaplan, 1995). The implication is that a general-purpose affect inventory can conceal the specific emotional signature of an esthetic encounter, so that individual and cultural differences in esthetic experience may be partly invisible when only broad affect dimensions are measured.

The third implication concerns the social and cultural basis of what counts as a desirable esthetic outcome. Affect valuation theory distinguishes the feelings people actually have from the feelings they ideally want to have, and shows that ideal affect is culturally shaped: East Asian and Chinese samples value low-arousal positive states such as calm more, and high-arousal positive states such as excitement less, than European American samples (Tsai et al., 2006; Tsai, 2007). In the PANAS framework, high positive affect is defined as a state of “high energy, full concentration, and pleasurable engagement,” whereas low negative affect is described as “a state of calmness and serenity” (Watson et al., 1988). The pattern observed here, a reliable reduction in negative affect without a reliable gain in positive affect, therefore describes movement toward calm rather than toward excitement. In a Chinese setting this may be the very outcome that viewers value most, so an esthetic experience that looks modest when judged by gains in high-arousal positive affect may nonetheless be well matched to culturally ideal affect. The instrument itself thus embeds culturally specific assumptions about which affective changes count as benefits, which matters for any study of the social basis of esthetic experience.

Cultural expectations about emotion and attention point in a similar direction. Esthetic experiences often blend positive and negative feelings, and models of art reception hold that negative feelings can be integrated into esthetic enjoyment rather than opposed to it (Menninghaus et al., 2017). A cultural tradition that expects change and tolerates contradiction (Spencer-Rodgers et al., 2010), and in which opposing feelings are expected to coexist (Miyamoto et al., 2010; Spencer-Rodgers et al., 2010), may make such mixed states easier to hold. This is consistent with the stable near-zero coupling observed here, although, as noted in Section 9.1, that coupling is also a property of the instrument and cannot be attributed to culture without a comparison group. Culture also shapes esthetic perception and preference directly, although not uniformly. East Asian viewers attend more to context and favor context-inclusive compositions (Masuda et al., 2008), and Chinese viewers rate traditional Chinese paintings more favorably than Western paintings while Western viewers show the reverse (Bao et al., 2016), whereas preferences for basic formal features such as symmetry, complexity, and contrast appear to be shared across cultures (Che et al., 2018). An enveloping installation, whether physical or virtual, in which the surrounding field is itself the work and viewers attend to the space as well as to the objects within it (Pelowski et al., 2018), may be especially congruent with a context-inclusive attentional style. The present single-culture data cannot test this possibility, which nonetheless identifies a concrete route by which the cultural background of viewers could shape their response to immersive art.

The fourth implication concerns the social setting of the encounter, which the present data could not observe but which the literature suggests is integral to it. Original artworks viewed in a museum are appreciated more and looked at for longer than reproductions of the same works viewed in a laboratory (Brieber et al., 2014), and esthetic evaluations shift in response to the lower ratings of other people and even to cues of being watched (Hesslinger et al., 2017). Immersive installations in public venues are usually visited in shared spaces, although the record for the present installation, which combined virtual reality with EEG recording, does not state whether participants experienced it alone or with others. Immersive virtual-reality presentation also intensifies awe relative to conventional two-dimensional viewing (Chirico et al., 2017). Awe diminishes the salience of the self and increases prosocial behavior (Piff et al., 2015), which suggests that the benefits of immersive esthetic experience may extend beyond the individual affective change measured here to social outcomes such as felt connectedness and generosity. A multi-level framework of how leisure and arts engagement affect health makes the same point more generally, distinguishing psychological, biological, social, and behavioral mechanisms that operate at individual, group, and population levels (Fancourt et al., 2021). The present study captured only the individual psychological level. The implication is that an affective change recorded on an individual questionnaire is one component of a socially situated experience, and that the circumstances of viewing, such as who was present, whether the experience was shared, and how others appeared to respond, may help explain why some visitors responded reliably and others did not.

These implications translate into specific directions for future research. First, studies should ask who benefits, by measuring the individual differences that the esthetic literature identifies as consequential, including openness to experience (Silvia et al., 2015; Fayn et al., 2015), art interest and art knowledge (Specker et al., 2020), and general esthetic responsiveness (Schlotz et al., 2021), together with age and baseline stress, and by testing whether these characteristics predict membership in the reliable-responder group identified here; detecting such moderation will require samples several times larger than the present one. Second, studies should broaden what is measured, pairing the PANAS with an instrument designed for esthetic emotions that also records calming effects such as relaxation (Schindler et al., 2017), adding items for low-arousal positive states such as calm, and assessing ideal affect (Tsai et al., 2006) so that the fit between actual and culturally ideal affect can be tested directly. Third, cross-cultural designs in which Chinese and Western participants experience the same installation, with dialectical thinking measured at the individual level (Spencer-Rodgers et al., 2010), would establish whether the asymmetry between negative and positive affect and the near-zero coupling reflect culture or the instrument, and varying whether the installation draws on Chinese or Western visual traditions would test whether cultural identity and context-inclusive attention shape the response (Masuda et al., 2008; Bao et al., 2016). Fourth, the social context of viewing should be treated as an experimental variable, comparing solitary viewing with viewing alongside friends or strangers and manipulating cues about other people’s reactions (Hesslinger et al., 2017), while measuring social outcomes such as connectedness and prosocial behavior (Piff et al., 2015). Fifth, combining *in situ* tracking of movement and attention (Pelowski et al., 2018) with experience sampling would connect moment-to-moment individual responses to the social encounters that occur during a visit. Together, these directions would move the question from whether immersive art changes affect to how individual dispositions, cultural values, and social situations jointly determine what an esthetic encounter does for a given person.

### 9.3 Practical implications

Practically, the results suggest where immersive art is most likely to help. Any practical reading must be tentative, because the study has no control condition, no demographic covariates, and a sample of only 30 people. With that caveat, the results are best treated as hypothesis-generating rather than as a basis for deployment. The observation that the reliable effect fell on the negative side of affect is consistent with the possibility that immersive art may be most relevant in settings marked by stress or tension, but this would need to be established in controlled studies before any programming or policy recommendation could follow. We therefore refrain from proposing specific campus interventions or cultural-policy measures on the strength of these data alone. The pattern of accumulating benefit over repeated engagement reported in the arts-on-prescription literature (Jensen et al., 2024) points to dose and repetition as variables that future controlled work should examine.

### 9.4 Limitations

Several limitations bound these conclusions. The design is a single pre-post measurement with no control condition, so the change cannot be cleanly separated from the passage of time, from repeated testing, or from expectancy. The sample of 30 is small, which limits statistical power, forces the structural equation models to a simplified parcel-level form, and rules out an item-level network analysis of affect coupling; the coupling question was therefore addressed through a dependent-correlation test rather than a formal network comparison. As a secondary analysis the study inherits whatever sampling and procedural features the original data collection imposed, and it lacks demographic covariates that might have contextualized the effects. Generalization beyond the specific setting, sample, and installation should be cautious. None of these limitations is hidden, and each points to a concrete improvement in future work.

### 9.5 Future work

Future work should add the controls this design lacks. A randomized comparison with an active control condition, such as a non-immersive art display or a neutral waiting period, would isolate the effect of immersion itself. Longitudinal follow-up would show whether the reduction in negative affect persists or decays, and experience-sampling during and after exposure would capture the moment-to-moment dynamics that a two-occasion design cannot. A larger sample would support item-level network models and a formal network comparison test of affect coupling, along with full scalar invariance testing. Replication across different installations and different Chinese subpopulations would establish how far the present pattern generalizes. Directions that bear specifically on the social and individual bases of esthetic experience, namely individual-difference moderators, culturally valued affect, cross-cultural comparison, and the social context of viewing, are set out in Section 9.2.

## 10 Conclusion

This secondary analysis of within-subjects PANAS data found that an immersive art exposure in a Chinese setting was followed by a reliable reduction in negative affect, no reliable change in positive affect, and no change in the near-zero coupling between the two dimensions. The conclusion held across mixed models, individual-level reliable change, and Bayesian estimation, and it survived a measurement-invariance check that ruled out response shift as an explanation. Read in terms of the social and individual bases of esthetic experience, the pattern suggests that the affective value of the encounter was person-contingent and took the form of movement toward calm rather than toward excitement, and it raises testable questions about how individual dispositions, culturally valued affect, and the social setting of viewing shape that value. The effect is modest and well estimated rather than large and fragile, which is exactly the kind of finding that a careful analysis of a small dataset should produce.

## Data Availability

The original contributions presented in the study are included in the article/Supplementary material, further inquiries can be directed to the corresponding author.
