# Supplementary material for: Affective dynamics following immersive art in a Chinese context: a secondary analysis of pre-post PANAS data
Source: Front Psychol. 2026 Oct 14;17:1941264. doi: 10.3389/fpsyg.2026.1941264 (PMC13713348; doi:10.3389/fpsyg.2026.1941264)
Supplement: Supplementary file 1 [file Data_Sheet_1.docx]

# **Supplementary Materials**

Algorithm S1 below gives the full computational pipeline in pseudocode. It is provided for reproducibility and is not required to follow the analysis in the main text.

**Algorithm S1.** *Triangulated pre-post affect analysis.*

```
input: item-level PANAS responses X[pre], X[post] for N respondents
1  load data; match respondent ids across pre and post
2  screen responses in range 1 to 5; flag straight-lining (within-person SD = 0)
3  form PA and NA composites = sum of 10 items per subscale per occasion
4  compute Cronbach alpha and McDonald omega per subscale per occasion
5  build 3 balanced parcels per subscale per occasion
6  for scale in {PA, NA}:
7      fit configural CFA; fit metric CFA (equal loadings across occasion)
8      invariance holds if |dCFI| <= 0.01
9  for outcome in {PA, NA}:
10     fit mixed model  Y ~ occasion + (1 | respondent)
11     compute dz and drm with bootstrap 95% CI (B = 10000)
12     compute RCI = (post - pre) / S_diff; classify Jacobson-Truax
13     fit parcel-level latent change score model
14     estimate Bayesian posterior of standardized change (4 chains,
        4000 draws, 2000 tune, target_accept = 0.95); decide via ROPE, BF
15 test change in PA-NA coupling: dependent-correlation Z (Steiger)
output: coefficients, effect sizes, reliable-change rates, posteriors,
        invariance verdicts, coupling test
```
